# An Intermediate in the evolution of superfast sonic muscles

**DOI:** 10.1186/1742-9994-8-31

**Published:** 2011-11-29

**Authors:** Hin-Kiu Mok, Eric Parmentier, Kuo-Hsun Chiu, Kai-En Tsai, Pai-Ho Chiu, Michael L Fine

**Affiliations:** 1 Institute of Marine Biology and Asia-Pacific Ocean Research Center, National Sun Yat-sen University, Kaohsiung 80424, Taiwan; 2 Laboratoire de Morphologie Fonctionnelle et Evolutive, Institut de Chimie, Université de Liège, - B6C, 4000 Liège, Belgium; 3 Department and Graduate Institute of Aquaculture, National Kaohsiung Marine University, 142 Hai-chuan Rd. Nan-tzu, Kaohsiung 81157, Taiwan; 4 Department of Biology, Virginia Commonwealth University, Richmond, VA 23284-2012, USA

**Keywords:** sound production, acoustic communication, swimbladder, striated muscle, smooth muscle, adaptation, evolutionary intermediates

## Abstract

**Background:**

Intermediate forms in the evolution of new adaptations such as transitions from water to land and the evolution of flight are often poorly understood. Similarly, the evolution of superfast sonic muscles in fishes, often considered the fastest muscles in vertebrates, has been a mystery because slow bladder movement does not generate sound. Slow muscles that stretch the swimbladder and then produce sound during recoil have recently been discovered in ophidiiform fishes. Here we describe the disturbance call (produced when fish are held) and sonic mechanism in an unrelated perciform pearl perch (Glaucosomatidae) that represents an intermediate condition in the evolution of super-fast sonic muscles.

**Results:**

The pearl perch disturbance call is a two-part sound produced by a fast sonic muscle that rapidly stretches the bladder and an antagonistic tendon-smooth muscle combination (part 1) causing the tendon and bladder to snap back (part 2) generating a higher-frequency and greater-amplitude pulse. The smooth muscle is confirmed by electron microscopy and protein analysis. To our knowledge smooth muscle attachment to a tendon is unknown in animals.

**Conclusion:**

The pearl perch, an advanced perciform teleost unrelated to ophidiiform fishes, uses a slow type mechanism to produce the major portion of the sound pulse during recoil, but the swimbladder is stretched by a fast muscle. Similarities between the two unrelated lineages, suggest independent and convergent evolution of sonic muscles and indicate intermediate forms in the evolution of superfast muscles.

## Background

Although neural circuitry for vocalization shares similarities between some fishes and tetrapods [1], little is known about the evolution of fish sonic mechanisms. Rather than a homologous syrinx or larynx, fish sounds are produced by an extraordinary diversity of mechanisms utilizing super-fast muscles that appear to evolve convergently [2,3]. In common between fishes and tetrapods, muscles act on organs derived from endodermal structures: swimbladder in fishes and trachea in tetrapods. Sonic swimbladder muscles can be extrinsic or intrinsic. Extrinsic muscles typically originate on the skull and insert on the swimbladder or a bony structure attached to the bladder. Intrinsic muscles, the less common condition, attach exclusively to the swimbladder wall and tend to be associated with prolonged tonal sounds [4-6].

Muscles can change insertions over evolutionary time [7], but how the process would start is unclear. Head muscles have migrated to the swimbladder multiple times, and all known examples are sonic except in the lion fish in which the muscle appears to manipulate the bladder for posture control [8]. Based on embryology [9] and parsimony, Fine and Ladich [10] speculated that extrinsic muscles preceded intrinsic ones because it would be simpler to move the insertion of an existing muscle to the swimbladder than to create a muscle de novo. In support of this idea, intrinsic sonic muscles and the sonic nerves in toadfish form in the occipital spinal cord, migrate and attach to the swimbladder [9].

Fish swimbladder muscles are often considered the fastest muscles in vertebrates [11]. Sound from fast sonic systems is produced as a forced rather than a resonant response, i.e. muscle cycle time (contraction and relaxation) and not the natural frequency of the swimbladder cavity, determines sound fundamental frequency [4,12,13]. For instance, the oyster toadfish routinely contracts its muscles at 200 Hz to produce a courtship call with that fundamental frequency and can follow an electrical stimulus at 400 Hz without tetanizing [12]. However, slow bladder movement does not generate audible sound [12,14]. Therefore, the evolution of superfast muscles has been a mystery since there was no clear role for slow muscles, and intermediates were lacking.

Recently, slow sonic swimbladder muscles have been discovered in carapids [15] and appear to occur in various ophidiiform fishes [16-20]; these muscles tetanize around 10 Hz [15]. The carapid swimbladder contains a thin fenestra near its anterior pole, which stretches during contraction of the slow sonic muscle. The contraction pulls the anterior swimbladder forward until it snaps back exciting sound production. *Ophidion rochei *has more complex sonic system with a bean-shaped rocker bone protruding from the forward wall of the bladder that is rotated in opposite directions by antagonistic pairs of sonic muscle [20]. Its sound pulses consist of two parts, potentially corresponding to opposite motions of the rocker bone. Consistent with slow muscles, the muscle contraction rate generates the number of sound pulses (one pulse per contraction) but not the frequency spectrum of the pulses as in fast muscles.

The pearl perch *Glaucosoma buergeri *Richardson is an advanced perciform teleost unrelated to ophidiiform fishes and possesses swimbladder muscles suggestive of sound production [21]. Its sonic anatomy is complex and mirrors many aspects of ophidiids, suggesting the presence of slow muscles. In this study we investigate the anatomy of sound production including ultrastructure of sonic muscles and electrophoresis of muscle proteins, describe sound properties and relate them to sonic anatomy, and posit an evolutionary scheme that for the first time allows for intermediates in the evolution of super-fast sonic muscles.

## Materials and methods

Pearl perch were caught between about 60 and 150 m of water on the bottom by hook and line about 100 km from Kaohsiung Harbor, Taiwan. Despite decompression, most fish were capable of producing sounds, indicating intact swimbladders. We evoked disturbance sounds in 6 fish (430 to 550 mm standard length) in a polystyrene tank (52 × 30 cm: water depth 20 cm) by gently touching the fish's abdomen. Sounds were recorded with a hydrophone (HP-A1 Burns Electronics) on to a Sony linear PCM-M10 recorder and analyzed with Avisoft (at least eight sounds per fish). Protocols were approved by the National Sun Yan-sen University Animal Care and Use Committee.

Animals were returned to the lab for dissection, histology and electrophoresis. Histology on the internal muscle fixed in 7% paraformaldehyde in phosphate buffer suggested it was a smooth muscle, and therefore electron microscopy was performed on another individual. Several small samples of sonic and epaxial muscles were fixed in 2.5% glutaraldehyde. Morphology was first observed in 6-7 μm sections stained with toluidin blue and then ultrathin sections were stained with uranyl acetate and lead citrate and examined with a JEOL JEM 11SX electron microscope.

We utilized (1) SDS-PAGE analysis combined with LC-MS/MS to probe the muscles for alpha transgellin, a smooth-muscle specific protein and (2) Western blotting for troponin T, a marker present in striated but not smooth muscle, and alpha (sarcomeric) actin.

### Nano-HPLC-MS/MS analysis

Approximately 0.1 g of the two sonic muscles, white trunk muscle and the swim bladder were individually homogenized with a Pro200 homogenizer (Pro Scientific Inc. Oxford, CT USA) in 0.07 M sodium phosphate buffer (pH 7.4) and centrifuged at 4°C for 20 min at 16000 *g *to remove the cell debris. Supernatant protein concentration was determined with the Bradford protein assay reagent (Bio-rad). Proteins were resolved by SDS-polyacrylamide gel electrophoresis (PAGE) performed on a 12% gel, and bands were visualized with silver staining. Specific bands were excised for identification, washed three times with 25 mM ammonium bicarbonate in water and 50% (v/v) acetonitrile (ACN) in 25 mM ammonium bicarbonate. Protein reduction was subsequently performed by incubating in 0.5 M dithiothrietol (DTT) for 1 h at 56 °C and then alkylating with 50 μL saturated iodoacetamide (IAA) for 45 min at room temperature in the dark. The gel sample was digested with 20 μg of sequencing-grade modified trypsin (Promega) with a sufficient volume of 25 mM ammonium bicarbonate to completely saturate the gel. The sample was incubated at 37 °C for an overnight digestion. Supernatants containing the peptides were transferred to siliconized 0.5 mL Eppendorf tubes. Remaining peptides in the gel pieces were extracted by incubation with 20 μL of 50% (v/v) ACN containing 5% (v/v) formic acid for 20 min. Extracted peptides were combined with those in the Eppendorf tubes, and nano-HPLC-MS/MS analysis was performed to identify tryptic peptides.

Extracted peptides were transferred to vials, and 8 μL of sample was fractionated by capillary reverse-phase liquid chromatography on a C18 microcapillary column (75 μm i.d. × 15 cm) coupled with an ion trap mass spectrometer (LCQ DECA XP Plus, ThermoFinnigan, San Jose, CA). The mobile phases were 0.1% (v/v) formic acid in H_2_O (buffer A) and 0.1% (v/v) formic acid in acetonitrile (buffer B). The gradient profile consisted of a linear 0% to 5% buffer B at 2 min and then progressed to 5% buffer B at 2 min and to 60% at 40 min. The samples were introduced into the mass spectrometer through an electrospray source with the application of a distal 1.6-1.7 kV spraying voltage, and the scan range of each full MS scan m/z 450-2000.

The proteins identified by MS/MS fragmentation spectra were searched against the NCBI Actinopterygii (ray-finned fishes) sequence database (version 20090616; 148434 sequences) with the MASCOT algorithm (v2.1.0, Matrix Science, London, UK). The mass search parameters were set: peptide mass tolerance, 1 Da; MS/MS tolerance, 1 Da; peptide charge, +1,+2, and +3; data format, sequence (DTA); Instrument, ESI-TRAP.; missed cleavage, 2; consideration for fixed modifications such as carbamidomethyl, and for variable modifications as deamidated and oxidation.

### Western blotting

Western blotting was used to verify the expression of selected proteins including troponin T and sarcomeric actin. Ten μg of protein from the various muscles and the swim bladder were mixed with SDS sample buffer and heated at 95°C for 5 min. Proteins were separated on a 12% SDS-PAGE and transferred to PVDF membranes (Millipore). The swimbladder was used as negative control. The membranes were blocked in a 5% nonfat milk solution for 1 h at room temperature and then probed with antibodies against troponin T (1:200 dilution, Santa Cruz Biotechnology) and against a monoclonal antibody to alpha sarcomeric actin (1:1,000 dilution, Abcam). The membranes were washed 3× with tris-buffered saline containing tween-20 (TBST) and incubated with secondary antibody in TBST/2% skim milk. Bound antibody was detected with the Enhanced Chemiluminescence System. Chemiluminescent signals were captured with the Fujifilm LAS 3000 system (Fujifilm). Duplicate experiments were performed at least 3×.

Because the swimbladder did not contain muscle actin, 10 μg of each sample examined by Western blotting were separated and visualized by silver staining to verify the protein integrity of each sample.

## Results

**Figure 1:**
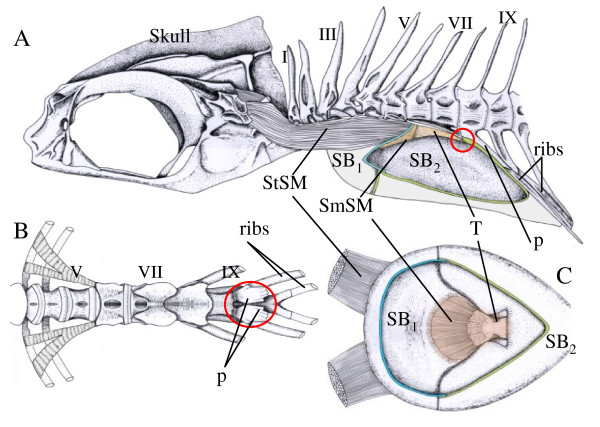
**Drawings of the sonic system of *Glaucosoma buergeri***. A. Lateral view of the sonic system. Latin numbers designate individual vertebra. B. Ventral view of the vertebral column illustrating processes that support the posterior swimbladder and attachment of the sonic tendon. The red circle indicates the insertion of the tendon on the paraphoyses of vertebra IX. C. Ventral view of the dorsal wall of the swimbladder (base of the swimbladder removed) illustrating the attachment of the tendon-smooth muscle to the inner tunica externa. Abbreviations: p parapohysis of vertebra IX, SB1 anterior part of the swimbladder, SB2 posterior part of the swimbladder, StSM: anterior striated sonic muscle, SmSM: smooth sonic muscle, and T tendon from vertebra IX to the smooth sonic muscle. The swimbladder fenestra, although not visible because it is covered by the tunica externa, is situated between SB1 and SB2.

The swimbladder, covered by a heavy white tunica externa, is broad anteriorly and tapers to a point posteriorly (Figure 1, animation in additional file 1). The posterior bladder is firmly attached to the hemal spine of the 9^th ^vertebra and to a ventral osseus plate made by the 7^th ^and 8^th ^vertebra and thus prevented from moving. The anterior pole of the bladder is unattached and free to move. The swimbladder fenestra occurs between the anterior and posterior regions of the bladder. It forms a dorsal slit that runs around the sides of the bladder to the ventral surface. The fenestra is covered by but not attached to the tunica externa of the posterior bladder.

Paired red anterior sonic muscles originate on the base of the pterotic bones on the skull and insert on the outside of the dorsal bladder at the forward edge of the fenestra (Figure 1). A tendon from the 9^th ^vertebra ends in a single red-colored muscle that inserts on the tunica externa on the inner side of the lumen of the anterior swimbladder. Therefore, the anterior striated muscle and posterior muscle-tendon spring apparatus attach to opposite sides of the bladder and appear to function as antagonists.

**Figure 2:**
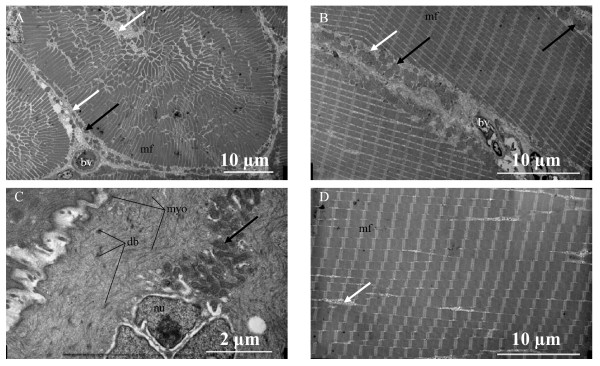
**Electron micrographs of sonic and hypaxial muscles of the pearl perch *Glaucosoma buergeri***. A. Cross section of a sonic fiber from the anterior-striated sonic muscle. B. Longitudinal section of a fiber from the anterior-striated sonic muscle. C. Longitudinal section of a fiber from the smooth muscle. D. Longitudinal section of epaxial trunk muscle. Note thicker myofibrils and thinner sarcoplasmic reticulum compared to sonic fibers. Abbreviations: bv blood vessel, db dense bodies, mf myofibrils, myo myofilaments in the smooth muscle, nu nucleus. Dark arrows indicate mitochondria and white arrows sarcoplasmic reticulum.

Electron microscopy indicates that the external sonic muscles are striated with a somewhat radial orientation in cross section and stacks of mitochondria at the fiber periphery but few in the interior (Figure 2A). Compared to epaxial fibers (Figure 2D), sonic fibers have thinner myofibrils and a more developed sarcoplasmic reticulum (Figure 2B, C), suggesting adaptations for speed [22] and fatigue resistance [4,23]. The internal muscle that connects the posterior tendon to the bladder has the morphology of a typical smooth muscle with dense bodies, myofibrils and a central nucleus adjacent to mitochondria (Figure 2C).

**Figure 3:**
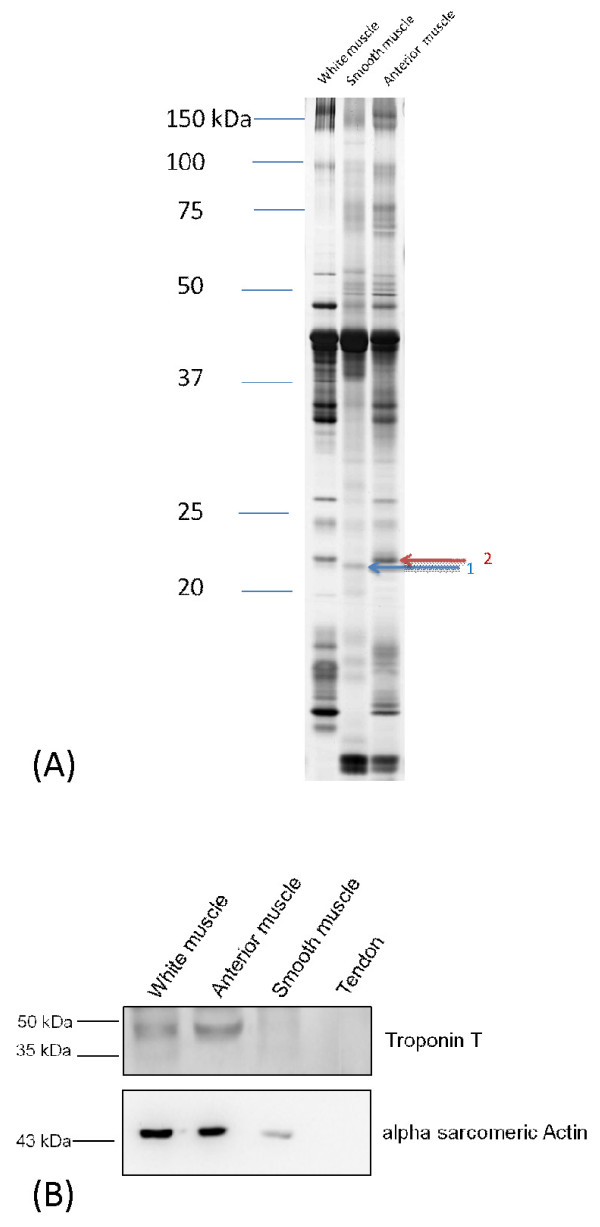
**SDS-PAGE (A) and Western blotting (B) analyses of *Glaucosoma buergeri *muscles**. Band 1, blue arrow: a protein homologous to the smooth-muscle specific protein transgelin, identified using LC-MS/MS followed by a database search, was revealed only in the smooth muscle. Band 2 red arrow: indicates myosin light chain 3 (see Additional File 2) from striated muscle (trunk white and anterior sonic muscle). B. Troponin T (upper panel) present in striated but not in the internal smooth muscle and alpha sacromeric actin (lower panel) present in muscles but not in the tendon. Actin, as expected, is expressed at a lower level in smooth than in striated muscles.

The smooth muscle-specific protein transgellin was identified in the smooth muscle but not in the anterior striated sonic or trunk muscle (Figure 3, Additional file 2). Additionally troponin T, typical of striated and heart muscle, was not present in the smooth muscle or the sonic tendon but was present in epaxial and anterior sonic muscle. Alpha actin was present in all three muscles but in lower concentrations, as expected, in the smooth muscle. The tendon served as a negative control, and no actin or troponin T were present although silver stained SDS-PAGE gels indicate abundant protein in the tendon. Therefore, electron microscopy and protein analysis identified the muscle attached to the tendon as smooth.

**Figure 4:**
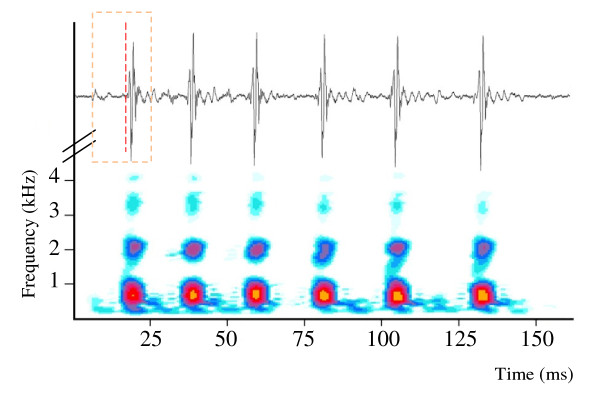
**Oscillogram and sonagram of a series of sound pulses evoked by touching the abdomen of the pearl perch *Glaucosoma buergeri***. The box in the oscillogram designates the first pulse, and the vertical dashed line separates pulse parts 1 and 2.

Disturbance calls consisted of a variable series of pulses (2-9) per call with a pulse period, or time from the beginning of one pulse to the next, of about 30 ms (means for individual fish varied from 24.7 to 32.6 ms) and a duration of about 120 ms (means varied from 86 to 198 ms) (Figure 4). There were several frequency bands and energy above 4 kHz, a rather high frequency for swimbladder sounds [2]. Each pulse consisted of two parts (pulse parts: PP1 and PP2). PP1 typically contained about 2 cycles, with each half cycle longer than its predecessor, indicating a forced rather than a resonant response. Note that cycle time would not change in a system primarily controlled by resonance). The period of the waveform, measured as the time between the two positive peaks, averaged 4.7 ± 0.3 ms (s.e.m.), equivalent to a frequency of 212 Hz (1,000 ms divided by pulse period). PP2 cycles were significantly shorter (1.9 ± 0.1 ms, paired t_5 _= 8.62, p = 0.0002), equivalent to a frequency of 540 Hz and exhibited several higher amplitude cycles before decaying. The amplitude of PP2 was also considerably greater than PP1 (calculated from voltage as 20 log V_PP2_/V_PP1_) by an average of about 17 dB (Figure 4), suggesting that PP2 is the more effective part of the call.

Pulling on the anterior sonic muscles extends the anterior bladder and fenestra forward and adds strain to the tendon-internal muscle combination, which pulls the bladder back upon relaxation of the anterior muscles. We therefore conclude that the weak PP1 is caused by contraction of a fast sonic muscle (the anterior striated muscles), and the higher frequency and greater amplitude of PP2 is caused by rapid rebound expected from a stretched tendon [24], as in slow sonic mechanisms.

## Discussion

The complex sonic anatomy of the pearl perch includes a number of structures that are uncommon in fishes [2]. The involvement of the smooth muscle is extraordinary. To our knowledge smooth muscles are not known to attach to tendons. Smooth muscle does occur within swimbladder walls as a thin layer [25] corresponding to the *muscularis mucosa *of the digestive tract. The red smooth sonic muscle is organized to assume a function at least partially analogous to that of striated muscle. Its precise function is not clearly understood, but slow speed of contraction is unlikely to contribute to sound frequency or pulse generation. In the syrinx of male ducks, smooth muscle is not the source of sound but has been hypothesized to modulate sound by altering tension of the inner tympaniform membranes [26]. Similarly smooth muscle contributes to a collagenous ring affecting gaze in primates [27]. For the pearl perch we suggest a common mechanical analog of a spring (the tendon) and a dashpot (the smooth muscle), which would function to damp swimbladder vibrations permitting pauses within a relatively rapid series of pulses (Figure 4) [28]. This hypothesis is supported by sounds we recorded from pempherids, a sister family to glaucosomatids. Pempherids have a tendon but no smooth muscle and produce longer duration pulses that continue to oscillate for more cycles before damping. The mechanics of contraction and rebound await study in these related families.

Although many but not most fishes have sonic swimbladder muscles [29], there is no known phylogenetic continuity as with the bird syrinx or mammalian larynx [1]. In typical fast systems in fishes, sonic muscles deform the bladder at a rapid rate that determines fundamental frequency [4,12,13]. Slow systems however, typically depend on rebound of stretched swimbladders and tendons [15-20]: features include a free anterior region of the swimbladder that is stretched by anterior sonic muscles, a stretchable swimbladder fenestra, a relatively fixed posterior region that is anchored to the backbone, and a tendon or other means of storing strain energy that causes the bladder to snap back rapidly. The fast sonic muscles of the pearl perch produce a weak component of the sound waveform suggesting that acoustic communication at any distance would depend on the second part of the pulse. The finding in pearl perch of a hybrid system, i.e. a fast muscle in a sonic system with the parallels to slow muscles listed above indicates a transitional form in the evolution of superfast sonic muscles. Therefore, we suggest a series of stages in sonic-muscle evolution: 1) no sonic muscles, 2) slow muscles that work primarily with a bone or tendon and produce sound by rebound, 3) fast muscles that still utilize rebound (i.e. the pearl perch and related families) and 4) fast muscles (extrinsic and intrinsic) that drive the bladder directly to express the peak frequency of the sound. Because swimbladder muscles appear to have formed independently multiple times, we suggest these stages would not necessarily correlate with the phylogenetic position of the family so that a more derived family could be at an earlier stage in the progression.

## Conclusion

The disturbance call of the pearl perch is composed of two parts. Although the swimbladder is stretched by a fast sonic muscle (part 1), the second and greater amplitude part of the call is produced by bladder recoil facilitated by strain energy in a tendon-smooth muscle pair. Rebound sounds are characteristic of slow sonic mechanisms in unrelated ophidiiform fishes. The pearl perch utilizing characteristics of slow and fast systems therefore represents in an intermediate condition in the evolution of superfast sonic muscles that drive swimbladder vibration directly.

## Competing interests

The authors declare that they have no competing interests.

## Authors' contributions

HKM initiated the study, verified sound production and performed dissections. EP performed electron and optical microscopy, made the morphological study and the drawings and analyzed sound files. KHC performed electrophoresis and proteomic analysis. KET collected specimens and recorded sounds. PHC analyzed sounds. MLF analyzed data and wrote most of the manuscript. All authors discussed the results, read and approved the final manuscript.

## Supplementary Material

Additional file 1 **Animation of the sonic anatomy of the pearl perch**. Click here for file

Additional file 2 **Table S1. List of identified proteins**. Table S2. List of homologues protein of protein 1 via a BLAST search. Click here for file
