# Supplementary material for: An Intermediate in the evolution of superfast sonic muscles
Source: Front Zool. 2011 Nov 29;8:31. doi: 10.1186/1742-9994-8-31 (PMC3251524; doi:10.1186/1742-9994-8-31)
Supplement: Additional file 2 — Table S1. List of identified proteins. Table S2. List of homologues protein of protein 1 via a BLAST search. [file 1742-9994-8-31-S2.PDF]

Supplementary data

Supplementary Table 1: List of identified proteins

Band number	Accession Number	Protein Name	Mw/pI	Mascot Score	Coverage Rate	Unique Peptide
1	gi|47224885	unnamed protein product	22.7/9.1	113	10%	M.AAKGVGMANK.G<br>R.TLSALGSLAVTK.D
2	gi|222087971	myosin light chain 3	19.0/4.47	263	38%	R.IVLSTLGEK.M<br>K.GTYDDYVEGLR.V<br>R.ALGQNPTNKDVNK.I<br>R.VFDKEGNGTVMGAELR.I<br>R.VGDSQVAFNQVADIMR.A

Supplementary Table 2: List of homologues protein of protein 1 via a BLAST search.

Accession Number	Protein Name	Total Score	Query Coverage	Positives	E-value
gi|309386893	transgelin 1	348	96%	185/197 (94%)	$2x\ 10^{-94}$
